# Exploratory expertise and the dual intentionality of music-making

**DOI:** 10.1007/s11097-019-09626-5

**Published:** 2019-06-18

**Authors:** Simon Høffding, Andrea Schiavio

**Affiliations:** 1 RITMO Centre for Interdisciplinary Research on Rhythm, Time and Motion, University of Oslo, Postboks 1133, Blindern, 0318 Oslo, Norway; 2 Centre for Systematic Musicology, University of Graz, Merangasse 70, 8010 Graz, Austria

**Keywords:** Expertise, Teleomusicality, Music-making, Enactivism, Performance, Consciousness

## Abstract

In this paper, we advance the thesis that music-making can be advantageously understood as an exploratory phenomenon. While music-making is certainly about aesthetic expression, from a phenomenological, cognitive, and even evolutionary perspective, it more importantly concerns structured explorations of the world around us, our minds, and our bodies. Our thesis is based on an enactive and phenomenological analysis of three cases: the first concerns the study of infants involved in early musical activities, and the two latter are phenomenologically inspired interviews with an expert jazz improviser, and members of a prominent string quartet. Across these examples, we find that music-making involves a dual intentionality - one oriented towards the exploration of the sonic, material, and social environment, and one oriented toward the self, including the exploration of bodily awareness and reflective mental states. In enactivist terms, exploration is a fundamental way of making sense of oneself as coupled with the world. Understanding music-making as a pre-eminent case of exploration helps us explicate and appreciate the developmental, sensorimotor, and more advanced cognitive resources that exist in music-making activities.

## Introduction

In this paper, we argue that music-making (i.e., performing, practicing, improvising, etc.) is an intrinsically *explorative* activity. It is explorative in several senses of the word: by playing a musical instrument, one can engage with the qualities of different sounds, discover novel ways to develop expressive musical nuances, and gain access to memories, emotions, imaginations, and meanings that were not available before the music-making act. One can also explore the wide range of physical movements necessary to achieve a particular musical intention - different fingerings, postures, and breathing patterns. Musical utterances can always be (re-)discovered, transformed, and negotiated on the basis of the contextual contingencies of the performance (Borgo 2005; Walton et al. 2014, 2015). Moreover, many forms of music-making are participatory and involve more than one agent. Here, explorative dynamics emerge as relational properties of the event: musical intentions, goals, actions, movements, and emotions depend on the interaction that gives rise to the music (Loaiza 2016; Schiavio & Høffding 2015; Schiavio et al. 2017a). Novices and experts, in various degrees, can explore their instrument, their ambience, their skills, and their emotions as the performance unfolds.

The present contribution aims at providing a more detailed phenomenological understanding of such complex activities by focusing on two particular kinds of explorative behaviors associated with music-making. On the one hand, drawing on developmental psychology, we consider how infants dynamically exploit the sonic properties of their environment and develop richer sensorimotor schemas that are relevant for their cognitive life. On the other hand, turning to musical expertise studies, we discuss how performing music can also drive an exploration of the musician’s own mind. By analyzing qualitative data generated with expert classical and jazz musicians,^1^ we provide novel insights into the complex development of, and interplay between performed music, motoric abilities, and structures of consciousness (e.g., perspectival self-awareness, time-consciousness, imagination, emotions, etc.). The two groups - toddlers and top-trained musicians - represent two extreme ends of musical life. We have chosen them because they bring out important structural similarities: playing music in all shapes and sizes (including performing with the voice, ‘non-musical’ instruments, and technologies), we claim, involves this dual exploratory feature, and hence develops one’s self-understanding and one’s coupling with the world. If toddlers and experts are united through their engagement in music-making as an exploratory activity, we have good reasons to assume that this also pertains to all music-making groups in between these two poles. This confers some degree of universality to the thesis, and advances our knowledge in the quest for understanding why there is, and always has been, music in all human societies.

Conceiving of music-making as an explorative activity is borne out of an enactivist framework in which action and perception compose a dynamic unity, and in which the mind is understood as a relational property of an organism coupled with its world (Varela et al. 1991). On this paradigm, cognition means making sense, not primarily as a detached, reflective capacity, but as a bodily grasping of the social and physical environment in which the organism is embedded (Di Paolo et al. 2017; Fuchs 2017; Thompson 2007). As a brain-body system in continuous interplay with its niche, the organism enacts rich patterns of action and perception that dynamically unfold and shape each other, revealing new properties of the environment that can be acted upon. We argue that music-making is a pre-eminent way to constitute, incorporate, and ultimately to make sense of such an emerging horizon of novel possibilities. What is at stake in the argument is the “dual intentionality of music-making”. The first one pertains to the rather trivial fact that when playing an instrument, we are usually directed at making music. The second is more contentious, and indicates that a covert intentionality is directed back at the “musicker” herself. As such, music-making becomes a means to explore one’s own bodily and mental capacities, as well as one’s environmental couplings in more or less systematic ways.

## Conceptual explorations

### Sensorimotor coupling

From the perspective of the living agent, perception never occurs in a vacuum. Rather, it is always embedded in a history of couplings that opens up a rich horizon of possibilities for action. A guitar, for example, displays different meanings and opportunities for the virtuoso guitarist and for the novice, respectively; similarly, a shaker affords a variety of experiences and behaviors to an infant versus an adult: while the adult does not need to touch and manually explore the object to understand its function and possibilities for physical engagement, the infant usually has to reach for and grasp it in order to become familiar with its complex structure and affordances. This drive for exploration allows novel motor repertoires to emerge, leading at the same time to the discovery of important properties of the object itself - its sonic features, or its hidden visual nuances.

In such cases, action and perception are equiprimordial - we perceive and immediately understand our environment because we can exercise our bodily knowledge (Nöe 2006; Merleau-Ponty 2002). Perceiving and doing become two complementary aspects of the same experiential dimension - an agent’s sensorimotor expertise shapes perceptual activity just like perception allows her to select the adequate motor patterns to guide behavior and further engage with the environment. In other words, through this action-perception coupling, new sensorimotor contingencies and perceptual structures emerge that lead the living system to meaningful perceptual and experiential discoveries.

### Exploration

Sensorimotor coupling lies at the foundation of the enactive approach (Di Paolo et al. 2017).^2^ We have presented the idea that perception is a form of action, but here we can rightfully ask, what kind of action? Gallagher claims that “perception is a pragmatic, exploratory activity” (Gallagher 2017, p. 41). In order to engage with our environment, we need our perceptual experience to be meaningful, we need it to make sense for us. And we do so by means of exploration - said differently, exploration is a condition of possibility of meaning-making. In the minimal sense of *the action of investigating an unknown area,* explorative behaviors allow agents to put forward their concerned “point of view”. Organisms are units in interaction that are constantly directed toward novel engagements to keep their own structural unity coherent and protected. Processes of “autopoietic regulation” have been found in bacteria exploring their niche, and ingesting a sugar gradient (Thompson 2007) - because sugar is the most significant nutrient for these organisms, they “cast a veil of significance” (Di Paolo 2009, p. 47) over their world to differentiate between encounters that are good or bad vis-a-vis their biological norms. More complex animals, like expert musicians, are also invested in exploring and producing a satisfying and meaningful musical performance. Here, the “gradient” is not sugar but, for instance, experiences of musical absorption or aesthetic satisfaction as well as novel form of interaction, as we shall go on to show.

### Sense-making

From the enactive perspective, the dynamic relationship between an organism’s internal (e.g., neural, metabolic, thermodynamic, etc.) biological processes, and the emergence of a meaningful world is understood as “sense-making” (Thompson and Stapleton 2009). Because this relationship is constrained by the complexity and affordances of both agents and their world, sense-making involves processes of skillful adaptation - or what Di Paolo and colleagues call “interactional asymmetry” (2017, pp. 124–139). The latter concerns the organism’s ability to regulate its own coupling with the environment, or its capacity to selectively integrate and generate perceptual and motoric experiences - those that are relevant to the moment-to-moment realization of all kinds of goals and social experiences of importance to its wellbeing. Understanding cognition in terms of the complex nexus comprising living systems (brain and body) and environment (social and physical) transforms the traditional view on mental life based on internal, computational models into a more relational one. Such a relational view of mentality allows for all kinds of extra-neural resources (cultures, material objects, technologies, and other living systems) to become part of one’s cognitive domain (see Malafouris 2013; Wheeler 2011). Given this externalist and non-individualist perspective, sense-making can also be understood as “participatory”, such that meaning and agency is generated and negotiated in social contexts (Cummins 2018; De Jaegher and Di Paolo 2007). Examples hereof can be found in studies on language (Cuffari et al. 2015), sport (Gesbert et al. 2017), dance (He and Ravn 2018), and indeed, music (Schiavio & De Jaegher, 2017; Krueger 2013; see also Torrance and Schumann 2018).

Music-making, in particular, consists of culturally mediated forms of skillful co-adaptations which involve a negotiation between the maintenance of an individual “perspective” or “point of view”, and the capacity to explore and develop novel experiences and strategies, depending on the context of the performance (Schiavio & van der Schyff, 2016; van der Schyff et al. 2018). Even when a particular piece or pattern is repeated over and over, we generally consider it to be an “exploration” over and above a “repetition”. A repetition does not capture the intrinsic dynamics and phenomenology of an exploration: consider how, in an oft-cited excerpt from Oliver Sacks’ *Musicophilia* (2007), the famous cellist Pablo Casals claimed that, at age 93, he had not grown tired of playing the entire Well Tempered Clavier by Bach on his piano every morning (having done so for more than 80 years). For him, each performance was an act of discovery where new beauty could be found. Casals’ example implies that, as musical practices are often based on repetition, it becomes essential to give new meaning to each musical engagement. Let us now examine how the concepts from these theoretical discussions “play out” in our empirical cases.

## Sensorimotor exploration and musical expertise

### Teleomusicality

Traditionally, theories of the ontogenetic emergence of music have focused on prenatal development and exposure (Parncutt 2009), caregiving and mother-infant interactions (Trehub and Trainor 1998), the infant’s perceptual abilities (Masataka 2006), memory (Saffran et al. 2000), movements (Phillips-Silver and Trainor 2005), singing behaviors, (Whiteman 2001), and cultural backgrounds (Soley and Hannon 2010). Other contributions, which align more with the aims of the present paper, studied infants’ capacity to explore by themselves their sonic environment in ways that are meaningful and goal-directed (Delalande 2009; Schiavio et al. 2017b; see also Krueger 2013). Consider again a situation where a particular object is placed in the visual domain of an infant stimulating her curiosity. Given its size, shape, and texture, the object can invite particular actions and modes of engagement (Corbetta and Snapp-Childs 2009). By investigating the object, the infant might discover its sonic properties and develop motor control to actively manipulate it: squeezing a toy might produce an unexpected sound that captures the toddler’s attention and excites her. This brings forth a new horizon of possibilities for sonic actions - and indeed young infants are often observed developing easy melodic and rhythmic patterns (Imberty 1995; Papoušek 1996), which can also involve social participation (see Custodero and Johnson-Green 2003, 2008). Infants do not always engage in these forms of exploratory behaviors by themselves; they are often encouraged to do so by a caregiver. This means that there are times where the infants’ exploratory activity becomes a triadic relation between themselves, the caregiver, and the emerging musical world.

In order to acquire information about the properties of the object, passive observation is often insufficient. It would leave the exploration incomplete in terms of audio-visuo-motor integration: many sonic properties of the object would remain undiscovered, its back side hidden, and the tactile sensation of holding it and playing with it completely unexplored. Bodily actions are a necessary resource for developing meaningful relationships with the toy and, in turn, establishing novel motor patterns, leading to even richer understandings of the object being examined.

When these kinds of explorations are based on a clear focus on the sounds generated, rather than, say, visual, motoric, or proprioceptive features of the toy, infants are involved in “teleomusical” behaviors (Schiavio et al. 2017b). That is, when adequate kinematic fluidity is developed and infants creatively play with sounds during their manual exploration of objects, a first musical environment is created. Such a musical activity differs in many ways from a developmentally earlier “protomusical” behavior - e.g., a vocalization exchange between infants and caregiver. It is defined as *protomusical* because the primary goal of the activity does not explicitly involve the exploration (and further elaboration) of sonic opportunities, even if sounds and music-like behaviors are present. Consider again infants-caregiver interactions based on vocal, expressive, and melodic communication. While such sonic structures are usually considered as inherently musical (see Trevarthen and Malloch 2000), it might be argued that their goal remains unclear: infants have good reasons to focus on non-musical parameters despite the sonic environment being developed within the exchange. For example, they might need to establish a more intimate emotional and social connection with the caregiver; they might seek nutrition or attention, etc. In contrast, teleomusical behaviors specifically target the musical properties of the sonic environment and involve the active production and elaboration of melodic and rhythmic variations through patterns of action that are selected for this specific purpose.

On this view, ‘music-making’ becomes subsumed under the category of ‘exploration’ - musical affordances enhance opportunities for actions that infants use to explore the sonic domain of their niche as well as their own motor capacity. Such interplay informs, motivates, and generates further explorations directed toward the world as well as themselves (in terms of development and sensorimotor control). This understanding of music-making as both a means and an end of exploratory behavior complements recent work by Krueger (2013) centered on the notion of “musical scaffolding”. Such scaffolding pertains to the role of music in infant cognition as “a tool for driving primitive but experientially rich forms of empathy” (Krueger 2013, p. 177). Our account is compatible with Krueger's, but our analysis focuses more on the role of music-making - rather than listening - in developing one’s motoric and cognitive skills: through musically meaningful explorations, infants develop novel repertoires of action that help them become more autonomous and active in exploring their surrounding environment. In other words, infants engage in acts of self-discovery every time they participate in exploratory musical activity. For example, they improve and extend their existing motor vocabulary by familiarizing with the set of actions necessary to play with the sonic properties of a simple toy. They can shake it, or bang it against a wall, optimizing their manual grip, and gaining fluidity in executing a wide range of actions. As described by Schiavio et al. 2017b, this allows them to manipulate more objects in increasingly sophisticated ways, facilitating various aspects of their musical activity (e.g., the selection of the adequate motor program).

As we have discussed, infants can bring forth their domain of meaning through an intuitive and creative form of music-making. Familiarizing with actions and their respective sounds has important consequences for the developing organism: for instance, it can help optimize existing engagements with sonic sources, allowing the prediction of an action’s auditory outcomes, and the recognition of possible dangers. It can also contribute to the flourishing of musical expertise and promote social interactions directed toward a musical goal. This includes forms of peer learning and joint improvisation that might be explored later in life (Schiavio et al. 2018a; 2018b), as well as collective forms of composition (e.g., Morgan 1998). The dynamic interactions with the social and physical environment that emerge through performance thus develop sets of affordances that are relevant to the developmental trajectory of the organism, and in doing so, they constitute an exploratory form of sense-making. Here actions, perceptions, and novel sonic environments are enacted and negotiated in light of the moment-to-moment contingencies of the ‘performance’ and the biological complexity of the agent (see Schiavio 2014; 2016).

While the musical activity of infants can be studied from different angles, we here want to emphasize the continuity between two main aspects that ground their experience. On the one hand, we have actions and behavior oriented to the exploration of their niche, which allow the infant to discover new (e.g., sonic) properties of their world and interact with them meaningfully. On the other hand, infants develop novel understandings of themselves (e.g., bodily awareness, motor control). These two aspects, comprising a *dual* form of intentionality, recursively shape each other and contribute to the flourishing of the infants’ musical life through the emergence of relevant expertise. The latter encompasses forms of social play among infants, as well as situations where adults (e.g., caregivers or educators) help the infants develop novel musical activities, which goes to show that the development of this dual form of intentionality is always socially embedded.^3^ The infants’ musical explorations increasingly develop when adequate motor skills are mastered, as they are for experts, and when goals thereby can be directed away from immediate behavioral outputs.

Having considered aspects of the initial stages of musical activity in infancy, in the following part of the paper we will explore expert musicianship. As we shall see in both individual and collective contexts, performing music involves an exploratory phenomenology that points to a different kind of sense-making when compared to young infants - beyond the obvious difference in terms of expertise.

### Torben Snekkestad: Improvisation as explorative negotiation

Torben Snekkestad^4^ is a well-establish jazz saxophonist who excels in free improvisation, a style with no explicit agreements about what is to be played. The following data derives from observations, several informal conversations, and a single formal interview of ninety minutes about his practice of joint and solo improvisation, and concern musical techniques adopted for surprising others and himself. We present this material because it highlights how expert music-making can be understood in terms of negotiation and exploration.

When one gets surprised or, as he describes, “when the rug is pulled from underneath one’s feet”, new musical and mental spaces can be explored, which are liberating and inspiring. Torben uses a central metaphor describing the work of improvising with others as a “negotiation of roles”. Rather than being primarily focused on aesthetic expression, Torben describes joint improvisation as a form of communication with his co-players, or a joint voyage or exploration^5^: “who sets out a course…who creates a sense of form, who binds things together? Who can allow herself to be rhapsodic and fabulizing? Who is static? If that’s what you want… who opens up musical contrast?”

Torben performs in free improvisation settings because he enjoys developing new musical material, and at the same time, improving as an artist:

There is a possibility to work very intensively with some parameters of interaction together with others…which on the spot generates new ways of regarding yourself as a musician and of opening the material. You have a set of musical materials that you’ve worked on and you’ve been nerding with some textures. Now you throw it into a setting where it is lit up and where more layers of reflection are added. It is acted upon. Again, it is self-developing. At this point you develop your material. Not alone, but in a setting so you can return and see “ahh, ok, it [the material] was also capable of this”.

This way of being together is of mental or almost spiritual value: “there are techniques that allow me to open this room and only have that focus and be present in that”. Consider now the following, where a similar point is made: “so there is an openness to create something unique right where you are. This is presence. It is like, maximized”. Through and through, Torben’s language emphasizes exploration. He wants to “open” and also “investigate” a room, which is musical and mental at the same time. He “finds places” he “had not planned for”, and “makes excursions” within them. Improvisation allows him to “reach these places” in the “landscape of possibility”.

Torben’s explorations enable a receptive undergoing and a submission to mental spheres that have to open themselves. In other words, he has to be open both to musical things happening and to his mind going into new zones and phases as led by the music, rather than simply trying to control or plan the musical unfolding. (Cf. Høffding 2019, chap. 10). As he puts it: “you may start in a controlled place and the move toward something you don’t entirely control and exactly then do the landscapes, that can do something, open up”. What these landscapes “can do” is again both musical and mental. You push out into the landscape, beyond your comfort zone, and in return you get pulled in: you are shown musical openings and possibilities beyond your individual imaginative capacity, where “it is lit up”. Torben possesses a range of skillful mental and physical techniques to manage these explorations. The latter must not become too repetitive, while at the same time should avoid random musical confabulations with no sense of direction. To negotiate between these two positions, Torben has practiced steering his time-consciousness, and even uses the Husserlian notions of “retention” and “protention”. He adopts these terms to describe how he can “lean” forward or backward into his musical intentions to maneuver between repetition and randomness. The free improvisation setting, however, is risky business. The places you move and are moved into, may be closed, stale, and incoherent. And indeed, “it can also go awfully wrong. There are many examples of improvisations that misfired”. This, however, only testifies to the exploratory nature of the endeavor. Were there no risk of failure and getting lost, it wouldn’t be very exploratory to begin with.

With respect to our explorative theses, the observations above can be summarized into the following three kinds of expertise: Torben’s music-making is firstly a *technical* kind of explorative expertise, probing how material can develop and flourish in novel ways. Here we find again the first leg in our thesis on dual intentionality, as this expertise targets the music performance; it is also an *intersubjective* kind of exploratory expertise, based on how he and his co-improvisers interact (Cf. Høffding & Satne under review); and, finally, it pinpoints a *mental* explorative expertise, the second leg of dual intentionality, in which he observes how the employment of mental acts, such as the stretching of his retention and pretension, shapes performance. The three kinds of explorations and their associated kinds of expertise dynamically interact and push each other in a large network-like structure that spans the reflective, meta-cognitive and the pre-reflective bodily (ibid.). There is a lot more to be described about Torben’s extensive practice, but the above suffices to establish the empirical ground for our explorative thesis. We will return to the three aforementioned explorative dimensions in order to integrate it with the findings in the next case-study.

### The Danish String Quartet: Exploring musico-mental places

The following analysis is based on qualitative investigations of the phenomenology of musical absorption with a world-class chamber ensemble called the Danish String Quartet (DSQ).^6^ Høffding has followed the DSQ for about eight years, and conducted about twenty hours of phenomenologically inspired interviews (see Høffding & Martiny 2016) to better understand the musical mind in performance (all summed up in Høffding 2019). The interviews were all directed at tracing the phenomenology of musical absorption – e.g., whether thinking interferes with coping, how musical experts coordinate complex joint actions, and whether even minimal pre-reflective self-awareness (Dreyfus 2013; Sutton et al. 2011; Zahavi 2005) could be lost in deeply absorbed performance. The ‘exploration hypothesis’ of the present paper did not motivate these interviews. In the following, however, we will point to a somewhat tacit dimension emerging from these data, indicative of a sophisticated kind of exploration. We present some of the more *unusual* forms of intense musical absorption and then address how, over time, the DSQ members seem to become acquainted with, and learn to maneuver between them. Their ability to do so can be understood as an exploration of the different mental places in which one can find oneself while making music. In other words, as with Torben’s practice, we find a dual intentionality directed at these mental explorations as well as at the musical performances.

First, a clarification. When talking about the unusual forms of musical absorption, it should be noted that ‘unusual’ has two distinct references. It firstly and simply means *rare*. Intense absorption in music is inevitably a question of being carried away by the music (Høffding 2019, Chap 10). This disposition, however, is not subject to one’s will or control, and only manifests in its strongest forms on vary rare occasions. How rare, differs from musician to musician, but the most intense forms of absorption only occur a handful of times every decade or so for the DSQ members. The other meaning of ‘unusual’ is *phenomenologically altered*: in brief, phenomenology tells us that people generally have a stable perspective on their experiential life, as given from their ‘here’, from the first-person perspective of my embodied subjectivity (Gallagher and Zahavi 2008). It also tells us that any act of consciousness implies (and is co-presented with) an act of self-consciousness - what Zahavi calls a “minimal self” (2005). The DSQ members have undergone experiences in which these two experiential structures, namely those associated with ‘perspectivalness’ and ‘the minimal self’ are arguably altered. These two are called “ex-static absorption” and “absorbed-not-being-there” respectively.

In “ex-static absorption”, some DSQ-members enter into a perspective on their performance and on themselves as if ‘floating under the roof’, or as a quasi ‘out-of-body’ experience. The violist, Asbjørn Nørgaard, has a label for it - “being in the zone” - and describes it as a detailed awareness of one’s own body, the audience, and the music flowing. Such experience is presented with disinterest and neutrality as if it isn’t really himself playing, but that he is merely observing the performance unfolding. He feels like he does not interfere or need to control his fingers and bowing, but as if his mere thinking, imagination, or intention expresses itself directly in the performance. He compares himself to an army commander (rather than a foot-soldier) or chess-player who, through a detached will power, can control everything around him. Needless to say, this is a highly pleasant and gratifying experience. Similar experiences are reported in (Hytönen-Ng 2013; Hurlburt 2011), showing that this is not an arbitrary example, but points to a central element of some forms of intense musical absorption.

What we call “absorbed not-being-there” is also backed up in the literature on and by artists (Bastian 1987; Meinertz 2008). This form of absorption is in many senses diametrically opposed to the ex-static form, and consists in the temporary loss or re-orientation of central cognitive faculties. The DSQ cellist, Fredrik Sjölin, reports that in this absorbed form, he has no awareness of what is going on while playing: he has no perspective on himself or on his surroundings, and no memory of what has happened. He speaks of it as a state from which he wakes up: it takes time to realize where he is and what has happened, like waking from a kind of sleep. Yet, like ex-static absorption, it is intensely pleasant both at the level of bodily sensations and emotions.

These are extreme cases in which the very architecture of our consciousness is momentarily changed. In the case of *ex-static absorption*, the point of view from which the experiential perspective is given, is, if not entirely, then partially or quasi, moved away from the body of the musician. In *absorbed not-being-there*, the pre-reflective self-awareness normally accompanying all mental acts, is altered, such that one is momentarily uncertain in ascribing a just past practice or performance to oneself.

In the beginning of Høffding's investigations with the DSQ, these experiences stood out as extreme occurrences entirely beyond individual control. About five years later, however, the DSQ members (having performed about twice as many concerts), report that they more and more often approach these zones of absorption and have a form of acquaintance with how to engage and maneuver between them. There are certainly individual differences between the four musicians, but with the increased routine, it is as if their basic mental position in the performance setting become more grounded, yielding a better possibility to explore mental movements towards, between, or away from the zones of intense absorption. Here is Asbjørn’s description answering to Author A’s question of what has changed in his grasp of the mental and technical dimensions of performing between 2013 and 2017:

“In general, many things are easier now. You can get used to many of these situations. It is more rarely that I end up in mental corners of which I cannot get out. We become more and more routined. The more often you’ve been in a tight position and found a way home, the better you know that way”.

“If you sense that it is not really working for the ensemble, then you perhaps have more tools to work back into something that’s functioning again. It can be at a simple practical level…. You close your eyes, which is a de facto loss of control, but which often carries good things with it, because you often become overcontrolling when things become problematic and then closing your eyes is like a statement because you force yourself to lose control and it often ends in something good, although it can go entirely wrong”.

“We have probably all become more of the central army commander that the lieutenants”.

“Actually, it feels like you can change incredibly fast between those different attributes contained in “the zone” playing. In one moment, you can see the helicopter perspective on the quartet and in the next moment then you can hear the resonance very, very distinctly. It is such an attentive moment, in which you can change between these different [aspects]….it is very flexible, very, very plastic all the time and I feel like I can enter and exit without losing the plasticity”.

*Have you had any “out of the ordinary” experiences since the last time?*

“No, the fundamental edges and corners of my experiences are actually the same. I have just become better at making sure I find myself in the right places”.

Mentioning the “helicopter perspective”, Asbjørn refers to the experience of seeing everything as apart from himself; his reference to the development from “lieutenant” to “army commander” has the same implication. We see that Asbjørn is developing an increasingly precise grasp of his own mind, such that he can foresee trouble, evaluate how to get out of it, in case it arises, and also that he can enter and exit different perspectives more or less at will. Finding himself in “the right places” refers to mental places, not the right finger or bow positionings. In other words, he has achieved a kind of mastery in which his technical ability and his mental “place” are somehow detached. He can broach the same musical passages from various perspectives, and explore the dynamic relationships between mental perspectives, his technique, and the musical expression. While not directly expressed in this way, we interpret Asbjørn to be engaging in a kind of exploration where the musical notes, though never exactly identical from performance to performance, are relatively stable, while the variation among mental states can be more extensive. While none of the other DSQ members speak of themselves as mastering various mental “places”, they all subscribe to a teleology where “being open” to intense absorption guides their striving. And even if they cannot control the emergence of intense absorption, they are gradually developing a sense of where and how to enhance this openness.

In other words, besides the overt dimension of the *musical* landscape, there is a tacit *mental* landscape to explore. Both of these are tied into the singular act of performance. The DSQ members know their way in and out of different mental foci and forms of self-awareness; and the ongoing evaluation of how and where to yearn for openness constitutes a training ground for self-exploration and self-experimentation that plays out in their collective music-making.

Having gone over these three different cases of music-making – infants, solo improvisation, and joint music-making – let us now discuss in what ways they instantiate an explorative intentionality, to further ground the hypothesis that all forms of music-making contain a dual form of intentionality.

## Dual intentionality in explorative expertise and infants

We have presented examples involving infants and expert musicians to show how a dual intentionality plays out differently in light of the “musicker’s” history of structural coupling with the environment. The dual intentional form, as we have seen, is thoroughly grounded in experts who have spent thousands of hours systematically and deliberately honing a specific skillset. Infants, however, are taking the very first steps on their musical journey. To present differences and lines of continuity between infants and expert musicians, in what follows we will look closer at the experts’ case, and elaborate on the aforementioned three kinds of musical expertise. We argue that these replicate – albeit in a much more sophisticated form – the basic dual form that we have seen in the infants’ case.

In the experts’ case, we can analytically distinguish between three different kinds of explorative expertise. We call them (i) “musico-technical expertise”, (ii) “interactive expertise”’, and (iii) “mental expertise”. They are all present in our two examples of expert musicians, though in different degrees.

The first kind of expertise is a *musico-technical expertise*, which pertains to how one performs, develops different techniques, and learns to master musical instruments (including the voice). For expert musicians, such as Torben and the DSQ members, this type of expertise is clearly necessary, though not sufficient, for their status as leading performers. It has to be integrated with other more tacit aspects that more decisively ground their musical life into ‘social’ and ‘mental’ opportunities for explorations. As we will see, the kind of musical ‘sense-making’ they develop is intrinsically dependent on all three kinds of expertise.

Secondly, we may talk of *interactive expertise*, which is to do with the kind of awareness the musicians have of one another. For the cohesion of a string quartet and for the successful interaction of a joint free improvisation, the ability to tune in with one’s co-players is as essential as musico-technical expertise. The ability of “tuning in” relies on sophisticated mechanisms of joint attention, but also on shared body schemas (Maravita and Iriki 2004) and shared emotions described more fully in Høffding & Satne (under review) and Salice, Høffding & Gallagher (2017). This kind of expertise is surely important to the DSQ, but it rarely features explicitly in the interviews - partially because the role of each instrument in a classical string quartet remains rather fixed. Even if dynamics, tempi, and expression vary from performance to performance, it is never up for grabs who plays what notes. In contradistinction, for Torben, the interactive explorations are in the forefront of his awareness, and consequently in the interview, because a major task of the free improvisation format is to discover ways of deciding who plays what. Music-making is a scaffold for pleasant and sophisticated ways of being together. In the infant cases, we see the musically directed playful interactions with the caregiver, that as Krueger points out, drives “forms of empathic connectedness” (Krueger 2013, p. 177). For expert musicians, the musical interactions also scaffold intense emotions, which, when successful, extends to audience elation.

The third kind of expertise emerging from our cases can be understood as *mental*. Here, Torben and the DSQ again seem to converge: they find themselves in different mental zones and have an unusual self-mastery concerning how to move between these. To a greater or lesser extent, they work under an implicit teleology that certain states are desirable or existentially meaningful. In a sense, all three kinds of expertise converge as means to this end. In the DSQ case, we only see this tacitly contained in the interviews, where Asbjørn for instance, achieves a greater stability and mastery of his mind while performing. In Torben’s case, the mental expertise is much more systematic as he purposefully employs different mental abilities of reflection and imagination, and works to manipulate phenomenological structures such as time consciousness, to move the music and set his mind in gear toward a “maximized presence”. Mental expertise is equivalent to what Stachó, in a recent special issue of *Musicae Scientiae* on virtuosity, calls “mental virtuosity” (Stachó 2018). He argues that the prevalent focus, especially in the world of classical music on “technical virtuosity” (Stachó 2018, p. 539), overlooks the musical mind and its “mental dexterity” (ibid.) that brings about beautiful performance. While we applaud Stachó’s emphasis on the mental dimensions of music-making, we find his analyses to be rather oriented toward the individual mind in performance, and thus lack the interactive component we also aim to bring forth.

The three kinds of explorative expertise are fully integrated in the act of music-making, yet some are more basic than others. Musico-technical expertise is an enabling condition of mental explorations. Without technical skills, too much attention would be devoted to the basics of playing for the ability of “sculpting the mind” during performance to emerge. In a regime of classical music, it seems that musico-technical expertise often precedes interactive expertise. But that need not be the case. In the free improvisation context, the musico-technical and the interactive kinds of expertise work in a dynamic relationship, reciprocally developing one another, such that performing with others and shaping the music leads one to engage in musico-technical explorations on one’s own and vice-versa. In turn, this leads back to mental explorations, where one can actively shape one’s mental places and develop experiences and feelings that then are only partially autonomous or individual.

The deliberately trained forms of explorative expertise are more sophisticated versions of the fundamental dual-aspect intentionality of music-making. Most obviously, mental exploratory expertise is aimed at developing the musician’s own consciousness, − including her sense of identity and selfhood, whereas musico-technical exploratory expertise develops her understanding of her instrument and the music being performed. The musico-technical kind of expertise, nevertheless, feeds back into the mental, because it plays an important role in training the musician’s physical capacities, and hence develops her sense of self as an embodied, skilled agent. Finally, interactive expertise can be conceived of as a janus-faced middle-player: the reciprocal action of adjusting-to-others-as-adjusted-to-oneself tracks both a heightened understanding of oneself and of the others.

In the infant case, we can identify early stages of the above-mentioned forms of expertise. Grabbing, banging, and exploring an object like a shaker helps develop a cross-modal correspondence of sensorimotor schemas: producing a certain rhythm is a basic form of musico-technical expertise, by which infants make sense of their contingent niche, their objects, their body, and their own abilities in shaping these. In this case, the musico-technical and the mental coalesce: the infant does not yet have the conceptual and autobiographical capacities to work deliberately on her sense of identity or self, but she does have a pre-reflective, embodied subjectivity. On this view, each teleomusical act strengthens a sense of skilled agency - a sense of being a situated creature whose relationships with objects and their sonic opportunities are meaningful. Moreover, we know that these kind of motor actions facilitate the development of the ability to predict the goals of another’s actions (see Kanakogi and Itakura 2011) and the understanding of audiovisual synchrony (Gerson et al. 2015) - two important elements of a richer mental exploratory expertise. Finally, as mentioned, teleomusical acts are often facilitated and encouraged by a caregiver. This can account for a basic form of interactive expertise, involving forms of social play where adults stimulate a wide range of explorative behaviors. A caregiver may place an object in front of the infant and motivate her to engage with it, helping her develop novel rhythmic and melodic patterns. Music-making can thus be seen as a cognitive tool that fosters a bidirectional and playful communication coupled with the development of skilled action.

## Conclusion: The dual intentionality of music-making

Before concluding, we want to meet the objection that our exploratory account is trivial and not specific to music-making at all. When engaged in a soccer match, do I not identically explore the world (my co-players, the ball, the opponents, etc.), my motor repertoire, and their relation? Do I not herein train my technical and interactive expertise? And in yoga, martial arts, or meditation do I not explore my bodily technical expertise as well as my mental expertise in sharpening my awareness and concentration? We grant that most, if not all human practices are explorative and develop some of the above-mentioned forms of expertise, but it is hard to point to any other that involve the full triplet of technical, interactive, and mental. Like music, it might be that some forms of games and contemplative practices can be found in all cultures, and have developed since the earliest of societies. Such games and contemplative practices, however, would in many cases involve music-making or music-like behavior (for games, think about the teasing melodies in hide and seek, of singing with rope skipping or hand clapping games; for contemplative practices, think of devotional singing, mantras or musical trancing ceremonies). Our argument was never that music-making is the only form of exploratory behavior, but exactly that it is a pre-eminent one because it relies on and develops more or less systematic and sophisticated forms of dual intentionality. Further, on a more orthodox phenomenological line, our claim to dual intentionality should not be confused with the general claim about the transcendental structure of subjectivity as always involving the dyad of intentionality and self-awareness (Zahavi 2005, Chap 1). *Qua* transcendental, no human activity can change this fact. In contradistinction, our claim is an empirical one targeting music-making as an essential human activity that develops our minds and bodies in a specific dual-fashioned way.

In conclusion, we have claimed that music-making can advantageously be understood as an explorative activity that involves a dual intentionality. One is directed towards the domain of musical objects, instruments, and other agents, allowing one to actively generate music and master relevant skills to explore the world one inhabits. The second is directed toward the more intimate domain of self-reflection and selfhood, and involves the exploration of those “mental landscapes” that one develops and manipulates when making music. This dual notion of musical intentionality is not to be associated with any form of dualism. It pinpoints two complementary aspects of music-making by emphasizing the developmental and phenomenological continuity and interdependency of both poles of experience. Rather than using one’s sensorimotor skills as means to performing music (the first orientation of the dual intentionality), music-making can also become a tool to explore one’s own mind (the second orientation of the dual intentionality), experimenting with entry into zones that could potentially be dissociative if not kept under strict control. When examining expert musicians, we should not be deceived by the impressive musical display. There are more hidden layers of expertise involved in music-making than what meets the eye, and we should appreciate how pervasive music can be in development, cognition, and social life. On our conception, music-making is a mental scaffold, a phenomenon to enact and extend one’s cognitive domain - what Patel calls a “transformative technology of the mind” (Patel 2010).

Our exploratory thesis fits into the discussion of the evolutionary gain of music (Cross 2003; van der Schyff & Schiavio, 2017). Music is pervasive in all cultures and existed since the beginning of humankind. One should therefore think that is has some biological or evolutionary function (Patel 2008). Our contribution stresses that music-making - in one and the same act - helps develop one’s grasp of the world, on the one hand, and of one’s own mind and body, on the other. The way music-making can expand one’s consciousness, (the latter of the dual forms of musical explorative intentionality) has to the best of our knowledge not been stressed in musicological literature so far, or only so when coupled with rituals involving trance (e.g., Becker 2004). The mental expertise of explorative musicality is a self-mastery oscillating between being carried away and exercising executive control, leading to great mental flexibility and resistance to stress. Though not a musician, the artist Robert Irwin would probably agree with us, in writing that: “to be an artist is not a matter of making paintings or objects at all. What we are really dealing with is our state of consciousness and the shape of our perception” (quoted in Noë 2000, p. 123).^7^ Our definition of three kinds of expertise in expert music-making also integrates different strands of literature that focus on only one of these: while Stachó (2018) emphasizes the mental, and Krueger’s work highlights the interactive dimension of music as a tool for action and self-regulation (2011, 2013), we fuse those two with the equally important bodily and technical expertise in order cash out an enactive conception of music-making. Our notion of dual intentionality aims to capture the fluid negotiation between, and development of, perception, action, and reflection, pointing to a complex nexus of phenomenological and motoric explorations that shapes one’s musical and cognitive life.

## Footnotes

1 Much of this data has been analyzed in publications such as Høffding 2019.

2 Between the different strands of enactivism there is discussion of how far you can get with sensorimotor coupling in explaining the more complex aspects of cognition. Thompson, Gallagher and Di Paolo all agree that Noë’s “sensorimotor enactivism” is insufficient for explaining for instance bodily self-awareness the sense of agency, not to mention abstract thinking (Di Paolo et al. 2017, p. 37; Thompson 2005, p. 417.; Gallagher 2017) Moreover, relative to Thompson, Gallagher and Di Paolo, Noë’s focus on sensorimotor coupling arguably plays down the social embeddedness of cognition – a point he is seemingly addressing in his current work on visual art and perception.

3 With such social embedding one might ask, if we are not actually describing a triadic form of intentionality. We, however, resist such a characterization, as embeddedness is hardly intentional in the sense of being “directed at”. The teleomusically engaged infant is overtly directed at the musical properties of a given object and, perhaps slightly more covertly, at her own motor-repertoire. Though situated in a social context, she is not intentionally directed at it.

4 http://torbensnekkestad.com/

5 Torben is surely not the only jazz musician who understands his practice through the language of exploration. Sudnow’s *Ways of the Hand* (2001), for example, is ripe with such references.

6 See: www.danishquartet.com

7 Irwin’s claim here calls attention to the fact that several thinkers describe different art forms and even aesthetic experience in language similar to our dual intentionality claim: for instance, in regard to painting, Wollheim (1987) writes of “twofoldness”, which, in the context of acting, is taken up by Smith (2011) and also by Gallagher and Gallagher (2019) who think of it as “double attunement”. In phenomenological aesthetics, we similarly find Dufrenne meticulously analyzing the co-dependency of aesthetic object and the subject for the emergence of aesthetic experience (1973). It seems worthwhile, yet beyond the scope of current article to compare and contrast these family resemblances.
